# Composted PBST Biodegradable Mulch Film Residues Enhance Crop Development: Insights into Microbial Community Assembly, Network Interactions, and Soil Metabolism

**DOI:** 10.3390/plants14131902

**Published:** 2025-06-20

**Authors:** Liuliu Li, Liyuan Liu, Guoyuan Zou, Xuexia Wang, Li Xu, Yong Yang, Jinfeng Liu, Huabo Liu, Dongsheng Liu

**Affiliations:** 1 Institute of Plant Nutrition, Resources and Environment, Beijing Academy of Agriculture and Forestry Sciences, Beijing 100097, China; liliuhb@126.com (L.L.); wxx0427@163.com (X.W.); 2 Institute of Environment and Sustainable Development in Agriculture, Chinese Academy of Agricultural Sciences, Beijing 100081, China; liuliyuan1001@163.com; 3 Institute of Quality Standard and Testing Technology, Beijing Academy of Agriculture and Forestry Sciences, Beijing 100097, China; xuliforever@163.com; 4 SINOPEC (Beijing) Research Institute of Chemical Industry Co., Ltd., Beijing 101111, China; yangy.bjhy@sinopec.com (Y.Y.); liujinf.bjhy@sinopec.com (J.L.); 5 Miyun District Agricultural Technology Extension Station in Beijing City, Beijing 101599, China; liuhuabo@163.com

**Keywords:** biodegradable mulch film (BDM), composting, crop growth, soil microbes, metabolites

## Abstract

Biodegradable mulch film (BDM) is regarded as a key solution to combat plastic mulch film pollution due to its ability to degrade completely into CO_2_ and H_2_O through environmentally friendly microorganisms. However, commercial BDM often fails to degrade fully after use, leading to the accumulation of BDM residues in soil and their transformation into microplastics (MPs) via various processes, posing a threat to the soil ecosystem. Given these discrepancies between the theoretical and practical degradation performance of BDM, there is an urgent need to understand the impacts of BDM residues on plant growth and soil health. This research conducted pot experiments spanning the entire growth cycle of Chinese cabbage to evaluate the impact of PBST-BDM raw material (R), PBST-BDM residues (M), and PBST-BDM composting product (P) on crop growth and soil quality. The findings revealed that R treatments had a slight effect on Chinese cabbage growth (e.g., a 5.80% increase in emergence rate in R 1% treatment, *p* < 0.05), while M treatments significantly hindered the emergence rate, plant height, leaf area, and biomass accumulation of Chinese cabbage by 30.4% (*p* < 0.05), 2.71 cm (*p* < 0.05), 39.0% (*p* < 0.05), and 1.86 g (*p* < 0.05) in the M 1% treatment compared to the control group (CK). In contrast, P treatments enhanced Chinese cabbage growth, with greater improvements at higher weight ratios, resulting in increases of 8.89% (*p* < 0.05), 4.96 cm (*p* < 0.05), 36.3% (*p* < 0.05), and 2.31 g (*p* < 0.05) in the P 1% treatment. Microbial network topology in the M 1% treatment is highly variable, with the increased proportion of positive correlations in the P 1% treatment hinting at stronger symbiotic interactions between species (*p* < 0.05). Analysis results of PCoA and PLS-DA showed significant differences in microbial community and soil metabolites between M 1% treatment and CK (*p* < 0.05). These findings suggest that, although composting post-use BDM may reduce their negative ecological effects, possibly via accelerating the early breakdown of residues, the feasibility and scalability of this approach require further validation under real-world field conditions.

## 1. Introduction

Considering the potential harm of polyethylene mulch film (PE) to ecosystems, biodegradable mulch film (BDM) has gradually attracted widespread attention [1]. In addition to biodegradable polyesters, the components/composition of BDM may include starches and celluloses, which enhance the biodegradability and reduce the cost [2]. In theory, BDM can decompose into harmless CO_2_ and H_2_O through the action of naturally occurring microorganisms within a specific timeframe after entering the soil. This decomposition process is likely facilitated by microorganisms that utilize enzymes to break down BDM polymer chains for energy [1]. BDM exhibits comparable efficacy to PE in maintaining optimal soil thermohygric conditions and suppressing weed proliferation [3]. Studies claimed that utilizing BDM not only increases rice yields and conserves irrigation water but also reduces greenhouse gas emissions from soil [4]. Moreover, due to its carbon-based nature, BDM enhances soil carbon storage through microbial breakdown, offering potential benefits for improving soil conditions and productivity [5].

Although BDM has many advantages, whether it can serve as a viable substitute for PE remains unclear. On the one hand, current methodologies rely on indirect parameters such as CO_2_ evolution measurements and macroscopic morphological observations [6]. Conventional testing is predominantly performed under idealized conditions, failing to replicate the dynamic characteristics of real-world agricultural ecosystems. On the other hand, commercial BDM does not degrade rapidly and completely after its lifespan. A study based on field data modeling predicts that it would require 21-58 months to achieve 90% degradation [7]. This implies that, while BDM is being transformed into CO_2_ and microbial biomass, microplastics (MPs, <5 mm) originating from BDM are produced. MPs can generate a series of negative ecological benefits in soil. For example, MPs reduce the cation exchange capacity of soil aggregates [8]. Given the heterogeneous characteristics of MPs and the adjacent soil, MPs can provide distinct habitats conducive to microbial colonization [9]. This enhances the transportation and enrichment of pollutants by MPs, resulting in a continuous deterioration in soil quality. Notably, MPs generated might migrate vertically down the soil profile through processes like soil water infiltration and/or biological disturbances, resulting in wider ecological impacts concerns [1]. However, extant research regarding their degradation remains comparatively sparse [10]. Given the ecological risks associated with BDM-derived microplastics demonstrated above, developing effective degradation pathways becomes imperative to mitigate secondary contamination.

From an ecological sustainability standpoint, composting is the most preferable choice for biodegradable plastics. Composting is a controlled process facilitated via microorganisms, commonly used to convert various residues into organic fertilizers. Studies indicate that composting enhances soil physical properties, boosts microbial activity, supports crop growth [11], and is integral to the processes of soil bioremediation and carbon sequestration [12]. The biodegradation of BDM under composting conditions seems to be a recommended approach, as most of the microorganisms involved in bioplastics degradation are typically found in soil or compost habitats [13]. Meanwhile, composting parameters have the characteristics of a nutrient-rich environment, well-suited humidity and temperature, and vigorous microbial proliferation and metabolic activity, all of which promote BDM decomposition. However, current research on BDM composting remains insufficient.

To address this knowledge gap, PBST material with superior properties (e.g., enhanced gas barrier performance) was selected as the research subject [14]. We investigate the differential effects and relationships of PBST-BDM raw material (R), PBST-BDM residues (M), and PBST-BDM composting product (P) on crop and soil, hypothesizing that the centralized composting of PBST-BDM may enhance its early degradation, thereby mitigating its long-term soil ecological impacts upon reapplication. The objectives include the following:

(1) Understanding the impact of PBST-BDM raw material, PBST-BDM residues, and PBST-BDM composting product on Chinese cabbage growth;
(2) Analyzing the varying responses of soil to these materials;
(3) Uncovering the relationships between different factors.

The findings will establish a basis for evaluating the safety of BDM farmland in China, promoting environmentally friendly, healthy, and sustainable cultivation strategies.

## 2. Results and Discussion

### 2.1. Growth Index of Chinese Cabbage

#### 2.1.1. Emergence Rates

**Figure 1:**
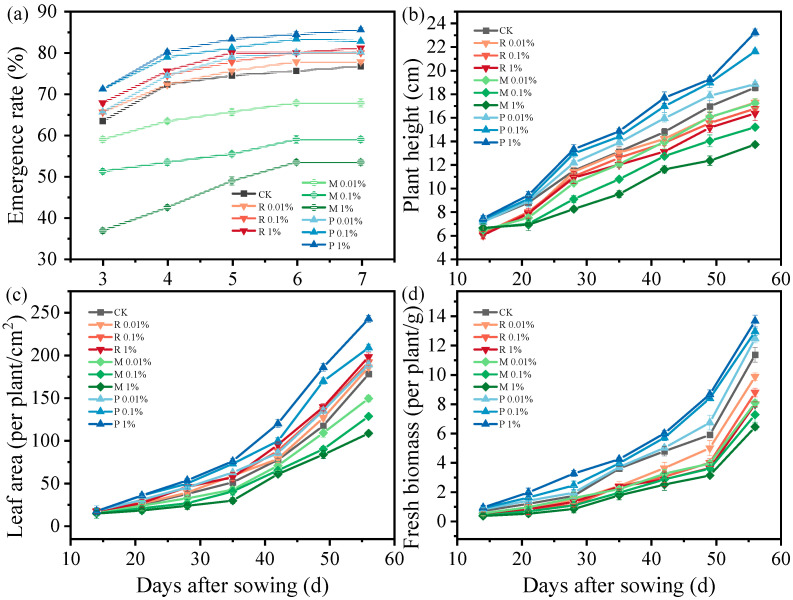
Time histories of emergence rate (**a**), plant height (**b**), leaf area (**c**), and fresh biomass accumulation (**d**) under different treatments for Chinese cabbage. R 0.01%, R 0.1%, and R 1% represent the weight ratios between PBST raw material and soil; M 0.01%, M 0.1%, and M 1% represent the weight ratios between PBST-BDM residues and soil; P 0.01%, P 0.1%, and P 1% represent the weight ratios between PBST-BDM composting product and soil. Significant differences in plant growth parameters among treatments (*p* < 0.05) are presented in Table S2.

The emergence rate of Chinese cabbage was investigated under treatments of PBST raw material (R), PBST-BDM residues (M), and PBST-BDM composting product (P), with three weight ratios (0.01%, 0.1%, and 1%) applied between each treatment (R, M, P) and soil (Figure 1a). Emergence rates consistently increased rapidly during the initial phases and slowed down during the final phases across all treatments. R treatment exhibited a slight impact on the emergence rate of Chinese cabbage, with a 5.80% (*p* < 0.05) reduction observed in the R 1% treatment compared to the control (CK). Obviously, the presence of PBST-BDM residues exhibited a notably unfavorable impact on the emergence rate. Higher concentrations of PBST-BDM residues led to a more pronounced inhibition of the emergence rate, aligning with previous research [15]. Studies indicate that residues from mulch film can alter soil porosity and air circulation, resulting in negative impacts on the sprouting of seeds and the growth of seedlings [16]. Water scarcity during the sprouting of seeds can result in decreased crop emergence rate. The infiltration rate of the soil was impacted by the residues from mulch film, leading to a restriction in water supply to crop seeds, and the degree of influence increased with the amount of residues [17]. Focus should be put on the problems that residues from mulch film can stick to the seed surface, inhibiting water absorption, or the seeds can become embedded in the residues, resulting in difficulty in penetrating the film and impeding seed germination [18], subsequently causing delays or decreases in germination rates. Consistent with previous studies, the emergence rate of Chinese cabbage under the M treatment decreased significantly by 11.6% (*p* < 0.05), 23.2% (*p* < 0.05), and 30.4% (*p* < 0.05) at residue weight ratios of 0.01%, 0.1%, and 1%, respectively, compared to the CK. In addition, acidic substances are released during the degradation of BDM, hindering the germination of seeds [19].

The use of PBST composting product significantly enhanced the emergence rate, showing a positive correlation with the weight ratio of PBST-BDM compost product and soil. The final emergence rate of Chinese cabbage under the P 1% treatment was 8.89% (*p* < 0.05) higher compared to the CK group. Composting can convert biodegradable materials into substances like humus, improving the quality and fertility of soil. Research has shown that composting can enhance the biodegradability of BDM, reducing its negative effects on soil or even benefiting soil health [20], thereby increasing emergence rates in this study.

#### 2.1.2. Plant Height, Leaf Area, and Fresh Biomass Production

Over time, the disparity in the growth indicators of Chinese cabbage among various treatments steadily increased (Figure 1b-d). These three growth indicators increased gradually across all treatments. PBST raw material slightly reduced plant height and fresh biomass accumulation (decreased by 2.2 cm and 3.37 g in R 1% treatment, *p* < 0.05), while it did not exert a significant detrimental impact on leaf area (increased by 11.3% in the R 1% treatment, *p* < 0.05). PBST-BDM residues significantly negatively affected the three growth indicators of Chinese cabbage, correlating with the amounts of PBST-BDM residues added to soil. In the M 0.01%, M 0.1%, and M 1% treatments (sampling at 7-day intervals), the decrease in plant height, leaf area, and fresh biomass accumulation were 0.9 cm (*p* < 0.05), 2.29 cm (*p* < 0.05), 2.71 cm (*p* < 0.05), 16.1% (*p* < 0.05), 28.0% (*p* < 0.05), 39.0% (*p* < 0.05), 1.59 g (*p* < 0.05), 1.22 g (*p* < 0.05), and 1.86 g (*p* < 0.05), respectively. Research conducted previously has confirmed the negative consequences of BDM residues on crops, such as reducing the plant height of wheat [21]. Crops can absorb MPs sourced from plastic film fragments’ degradation, modifying plant (particularly for root crops) root traits and influencing biomass accumulation [22]. In addition, decreased density parameters and increased soil porosity caused by MPs may impact crop development and the uptake of water and nutrients indirectly, which in turn affect root growth [23]. Modifications in the soil environment influenced microbial composition and functionality, which affect soil fertility and rhizosphere processes, thus impacting crop development [24].

After PBST-BDM composting, the plant height and fresh biomass accumulation markedly rose with a greater increase observed in higher proportions of soil. The increase in plant height and fresh weight were 0.58 cm (*p* < 0.05), 3.36 cm (*p* < 0.05), 4.96 cm (*p* < 0.05), 1.11 g (*p* < 0.05), 1.57 g (*p* < 0.05), and 2.31 g (*p* < 0.05) in the P 0.01%, P 0.1%, and P 1% treatments (sampling at 7-day intervals), respectively. The leaf area also expanded with the augmentation of PBST composting concentration, with an increase of 6.24% (*p* < 0.05), 17.3% (*p* < 0.05), and 36.3% (*p* < 0.05) in the P 0.01%, P 0.1%, and P 1% treatments, compared to the CK. PBST-BDM composting enhanced nutrient cycling, improved soil structure, and promoted plant growth, including height and fresh biomass accumulation. Studies have shown that some BDM can be composted and degraded to produce biomass [25]. The elevated temperatures and humidity levels in composting processes not only stimulate microbial metabolism and create optimal conditions for microbial proliferation, but can also affect the oxygen diffusion rate within the composting system. These conditions collectively contribute to accelerated oxidative decomposition of MPs through synergistic effects, thereby promoting their degradation [26]. Research has reported composting significantly enhances MPs’ biodegradability by reducing their abundance and particle size and influencing the microbial composition, thereby influencing MPs’ degradation process [26]. A significant reduction in MP quantity in sludge was captured when altering composting conditions and causing large-scale MPs to break down into smaller pieces, thereby accelerating their further degradation [27]. The adverse influences of MPs were reduced after composting in the present research, and the nutrients produced facilitated the growth of Chinese cabbage.

According to the present study results, the composted BDM showed a positive impact on Chinese cabbage emergence and growth compared to the untreated BDM. It is speculated that non-composted BDM left in the soil as a more active carbon source may be rapidly degraded and utilized by microorganisms in the short term. This microbial activity may lead to temporary anaerobic conditions and nitrogen depletion in the rhizosphere soil, creating changes in the micro-domain environment that are unfavorable for crop growth. The essence is that compost application to the rhizosphere and compost-plant interactions selectively recruit microbial taxa, leading to the short-term restructuring of the rhizosphere microbiome composition. Furthermore, although short-term experiments (covering the entire crop growth cycle) provide valuable insights into immediate effects, future research should prioritize investigating the long-term accumulation of BDM residues in soil and their potential impacts on crop productivity and soil ecosystem stability.

### 2.2. Variations of Soil Physicochemical Properties

**Figure 2:**
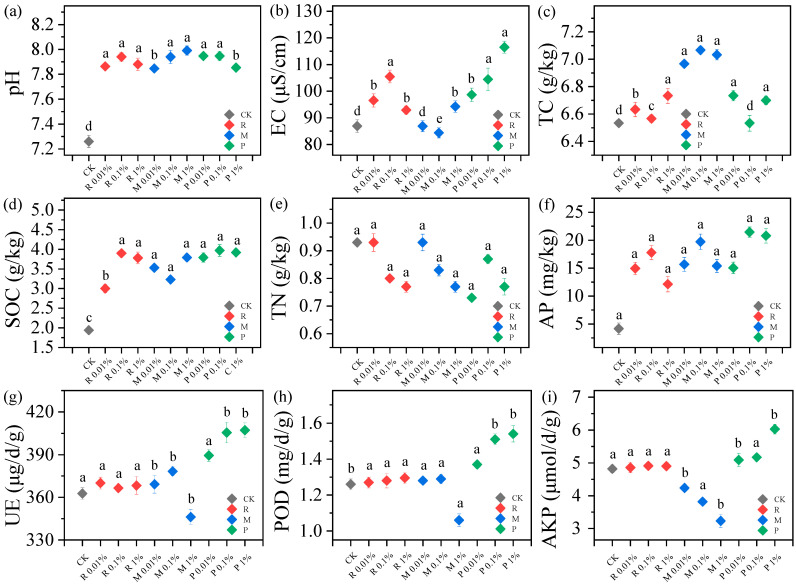
Variations of soil physicochemical properties and enzyme activities. Different lowercase letters (a, b, c, d, e) indicate significant differences among groups at the *p* < 0.05 level (one-way ANOVA followed by Tukey’s HSD test).

Six soil physicochemical properties (pH, EC, TC, SOC, TN, AP) and three enzyme activities (urease (UE), peroxidase (POD), and alkaline phosphatase (AKP)) were selected, as they characterize basic soil properties, fertility status, and microbial metabolic activity and serve as indicators for assessing pollutant-induced soil degradation. As shown in Figure 2, all treatments displayed a notable increase in pH relative to the CK. Within each group, soil pH showed no significant differences. The pH changes in the M treatments were evident, with M 1% treatment showing a 3.25% (*p* < 0.05) increase compared to the CK. Theoretically, the mineralization process of BDM might contribute to a decrease in soil pH via the organic acids produced [28]. The rise in pH in the M groups could be due to the presence of BDM inhibiting the growth of certain acidic microorganisms, as well as the differences in type, dosage, size, and exposure time. Enhanced microbial activity, which accelerates the decomposition of organic matter under high pH conditions, contributes to nutrient supply and cycling [29].

As a crucial indicator of soil physical properties, reducing EC poses a challenge to sustainable agriculture [24]. As shown in Figure 2, the R 0.01%, R 0.1%, and R 1% increased EC by 11.10% (*p* < 0.05), 21.40% (*p* < 0.05), and 6.90% (*p* < 0.05) respectively, possibly due to the release of additives. The M 0.01% treatment reduced EC by 0.03% (*p* < 0.05), while the M 0.1% treatment initially decreased it by 2.88% (*p* < 0.05), both possibly due to adsorption on soil colloid surfaces reducing free ion concentrations. However, the M 1% treatment ultimately increased EC by 8.44% (*p* < 0.05), likely caused by ion release from microbial metabolites and desorption processes on these surfaces [24]. Compared with the CK, the composting treatments (P 0.01%, P 0.1%, and P 1%) significantly increased soil EC by 13.5%, 20.2%, and 34.1%, respectively (*p* < 0.05 for all). PBST-BDM composting enhances ion exchange through organic acids or functional groups that modify the surface charge of soil particles. Concurrently, it stimulates microbial activity, leading to the release of inorganic ions via metabolites (e.g., organic acids) and enzymatic reactions. These combined processes elevate soil EC. A moderate EC increase further promotes microbial activity, improves nutrient transformation and release, and supplies crops with more bioavailable nutrients, thereby supporting growth [30]. However, excessively high EC levels may induce osmotic stress in microorganisms, ultimately inhibiting their proliferation. pH and EC can directly or indirectly affect soil nutrient absorption, thereby affecting soil fertility and ultimately impacting crop growth and development [30].

Both TC and SOC in the soil increased in the M treatments, which rose by 7.65% (*p* < 0.05) and 95.4% (*p* < 0.05) in the M 1% treatment, respectively. BDM residues in the soil can serve as an organic carbon source after degradation by microorganisms. While BDM contributes minimally to TC input, it stimulates microbial activity, potentially altering SOC dynamics. Microorganisms prioritize metabolizing BDM-derived carbon, partially converting it into SOC and transiently elevating DOC levels [31]. However, long-term SOC retention is constrained by environmental factors [30]. The R 1% and P 1% treatments significantly increased TC (3.06% and 2.55%, *p* < 0.05) and SOC (94.8% and 102.1%, *p* < 0.05), respectively. Through stimulating microbial activity, PBST-BDM composting facilitates the conversion of organic matter into stable humic substances, leading to a significant accumulation of SOC. Treatments P, post-composting, are nutrient-rich, boosting soil SOC and AP while slightly decreasing soil TN. The impact of the R and M treatments on TN levels was insignificant, in line with Liu’s results [3], but both increased AP.

The analysis of soil enzyme activity revealed that low levels of BDM residues (M 0.01% and M 0.1%) slightly increased UE by 1.79% (*p* < 0.05) and 4.30% (*p* < 0.05), respectively, and POD activity by 1.59% (*p* < 0.05) and 2.38% (*p* < 0.05), respectively. In contrast, the M 1% treatment caused significant reductions in both UE (4.50%, *p* < 0.05) and POD (15.9%, *p* < 0.05) enzyme activities. In the M 1% treatment, AKP enzyme activity was reduced by 34.1% (*p* < 0.05). Low concentrations of BDM may stimulate microbial activity, enhancing carbon cycling and thus improving soil carbon storage. In contrast, high BDM concentrations can compromise soil structure, reducing air permeability and affecting microbial growth and metabolism, thereby negatively impacting soil enzyme activity [30]. The released additives may interfere with the structure and function of the enzymes. After composting, the activities of UE, POD, and AKP increased significantly, with respective increments of 12.3% (*p* < 0.05), 22.2% (*p* < 0.05), and 25.1% (*p* < 0.05) under the P 1% treatment. The compost likely activated soil enzymes by providing abundant nutrients and improving soil physical properties [32]. Soil enzyme activity is closely related to the synthesis and regulation of plant hormones, and its changes can affect the balance of plant hormones, thereby affecting crop growth [29,30].

### 2.3. Soil Bacterial Community Dynamics in Response to Different Treatments

#### 2.3.1. Diversity and Structure

**Figure 3:**
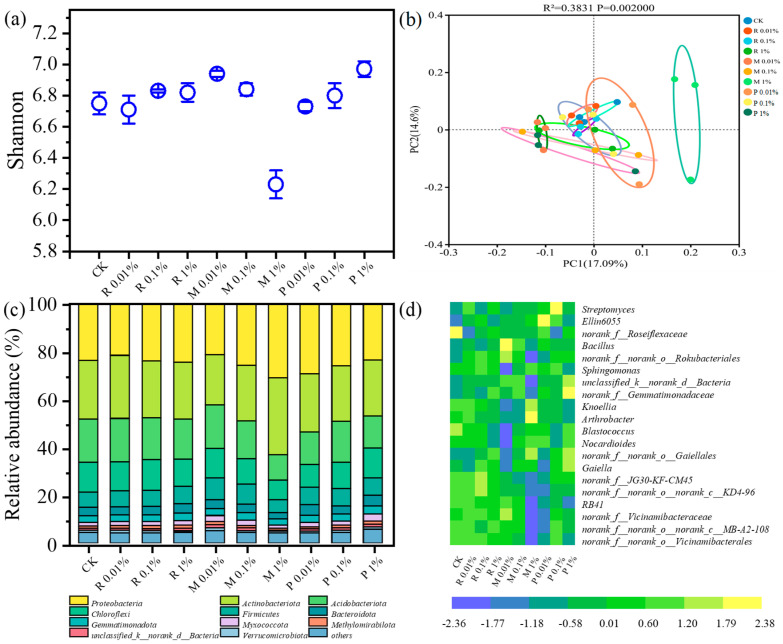
Effects of different treatments on bacterial community characteristics: Shannon index (**a**), principal component analysis (**b**), relative abundance on phylum level (**c**), and genus level (**d**).

Soil microbes are pivotal in the processes of nutrient cycling and biochemical transformation, altering the soil habitat through various mechanisms. Microorganisms deploy a variety of adaptive strategies to manage environmental changes. Figure 3a shows changes in microbial diversity under different treatments. Differences from CK were not statistically significant in the Shannon index of R 0.01%, R 0.1%, and R 1% treatments for microbial communities (e.g., a 1.03% increase in R 1% treatment compared to CK, *p* < 0.05). Notably, compared to CK, the Shannon index of soil bacteria in M 0.01% and M 0.1% treatments showed no significant variation, whereas a marked reduction of 7.7% (*p* < 0.05) was observed in the M 1% treatment. The understanding of how residues from mulch film affect soil microorganisms is currently inconclusive. For instance, some researchers reported that the diversity of bacterial taxa was not influenced when 10% BDM (*w*/*w*) was added [7], while others reported a reduction in the Shannon index when 1% BDM was added (*w*/*w*) [33]. Various factors such as the type of soil and mulch film, amount of mulch film residues, cultivation environment, and duration may all contribute to different outcomes. The Shannon diversity index increased by 3.3% (*p* < 0.05) with the P 1% treatment composting, indicating that composting enhanced microbial community diversity. Composting can alter soil physical properties, sustain soil fertility, and positively influence soil microorganisms [11]. Marked disparities were noted in the bacterial composition using the PCoA method at the OTU-level (Figure 3b). Differences were noted among various treatments, with explanatory variances of 17.09% (*p* < 0.05) and 14.6% (*p* < 0.05), respectively. High similarity was observed among samples within each group, with significant dispersion between groups. The clustering distance of the M 1% treatment is the farthest from CK, highlighting the greatest disparity between the two experimental conditions.

The phylum-level allocation of soil bacterial populations is shown in Figure 3c. *Actinobacteriota* (21.0–32.1%, *p* < 0.05), *Acidobacteriota* (10.5–18.3%, *p* < 0.05), *Chloroflexi* (8.29–12.5%, *p* < 0.05), and *Firmicutes* (5.28–9.02%, *p* < 0.05) are the top-four dominant bacterial phyla in all treatments. Compared to CK, the relative abundance of *Proteobacteria* and *Actinobacteriota* in M 1% treatment increased by 30.4% (*p* < 0.05) and 31.4% (*p* < 0.05), respectively, indicating their tolerance to BDM loading. This result aligns with Liu’s research, where the abundance of these two dominant phyla also increased [7]. These phyla have been found to contribute to the decomposition of MPs and carbonaceous substances in soil [34], which is beneficial for MP degradation. However, *Acidobacteriota*, *Phloroflexi*, and *Firmicutes* decreased by 41.8% (*p* < 0.05), 33.6% (*p* < 0.05), and 15.5% (*p* < 0.05), respectively, suggesting their sensitivity to BDM. For P 1% treatment, the abundance of *Firmicutes*, *Bacteroidota*, *Gemmatimonadota*, and *Myxococcota* increased by 14.6% (*p* < 0.05), 25.7% (*p* < 0.05), 22.3% (*p* < 0.05), and 1.09% (*p* < 0.05), respectively (compared to CK). *Firmicutes* are identified as the dominant bacteria in composting process [35]. Figure 3d further illustrates the microbial structure at the genus level. Across all treatments, *Arthrobacter*, *Sphingomonas*, and *Bacillus* are the principal genera. The proportional representation of *Arthrobacter* and *Sphingomonas* in M 1% treatment increased from 4.87% (*p* < 0.05) and 4.71% (*p* < 0.05) to 8.59% (*p* < 0.05) and 5.28% (*p* < 0.05), respectively, suggesting they may be the functional microorganisms associated with BDM loading. *Arthrobacter* and *Sphingomonas* have been reported to enrich on BDM, suggesting their potential as BDM degraders [36]. Known for degrading polymeric compounds, *Arthrobacter* was observed to have a significant enrichment [37]. *Sphingomonas* has the capability to degrade various harmful compounds, as shown in previous research [38]. The enrichment of these taxa may be associated with their ability to utilize BDM residues as the carbon source, through which *Arthrobacter* and *Sphingomonas* participate in the degradation of PBST-BDM and the mineralization processes of its degradation byproducts (e.g., MPs). A higher abundance of *Bacillus* was found in R 0.01% (3.78%, *p* < 0.05), R 0.1% (3.51%, *p* < 0.05), R 1% (4.05%, *p* < 0.05), P 0.01% (4.44%, *p* < 0.05), P 0.1% (3.95%, *p* < 0.05), and P 1% (3.93%, *p* < 0.05) compared to CK (3.25%, *p* < 0.05). The *Bacillus* genus, which belongs to *Firmicutes*, shows antifungal and competitive abilities [39]. The presence of *Bacillus* can enhance the humification effect during composting [40]. Additionally, *Bacillus* participated in the decomposition of biodegradable plastics [41]. *Bacillus* likely mediates BDM biodegradation during composting, concomitantly enhancing humification, which collectively improves Chinese cabbage growth and soil quality in the present study.

Soil microorganisms are highly responsive to environmental changes, as microbial communities tend to shift along with alterations in soil properties. The presence of BDM residues results in fluctuations in the abundance of microbial communities at both the phylum and genus strata. A significant shift in microbial community structure was observed in the M 1% treatment, indicating that the effects of MPs on soil microbial populations are connected to the amounts of residues present. The types of microorganisms experiencing significant abundance changes post-composting differ from those in the M treatment, highlighting the distinct adaptability of microorganisms to varying environments. Additionally, microbial diversity was enhanced under the P 1% treatment, further supporting the positive effects of BDM composting. More comprehensive exploration of the microorganisms involved in BDM composting is necessary to gain insights for the improved utilization of BDM.

#### 2.3.2. Examination of the Topological Characteristics of Co-Occurrence Networks in Bacterial Populations

**Figure 4:**
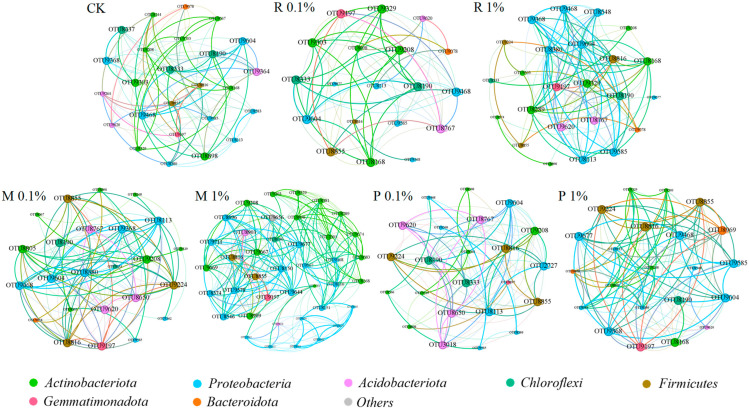
Co-occurrence network of soil bacterial community based on correlation analysis. Networks were constructed at operational taxonomic unit (OTU) level. The sizes of nodes (OTU) were scaled to the degree of nodes and the nodes were colored on phylum level. The edges colored by red and green represent positive and negative correlations, respectively. The significant difference between groups based on network topological indices (*p* < 0.05).

Network analysis can investigate how external stress affects the stability of microbial communities. The dynamics of symbiotic networks (such as changes in complexity) are often a response mechanism of soil microbes to external disturbances. Persistent environmental stress can decrease the network stability of microbial communities. A symbiotic network (OTU-level) analysis of soil microbial consortia was carried out (Figure 4 and Figure S2, Table S3). The dominance of positive correlations in all groups demonstrates that microorganisms are chiefly characterized by synergistic effect. Relative to CK, R 0.01% and R 0.1% treatments exhibited a decrease in both node and edge numbers, suggesting a diminished connectivity within the bacterial community. An increase in edges within the R 1% treatment shows that interactions between nodes have become more abundant and intensive. The alterations in node and edge counts in M 0.01% were insignificant. The increased number of edges in the M 0.1% treatment suggests that species exhibit more extensive symbiotic or interactive relationships under 0.1% PBST-BDM residue addition. The M 1% treatment resulted in a considerable increase in node and edge counts, while the proportion of positive correlations decreased, suggesting that the presence of 1% PBST-BDM residues increased network complexity and microbial interaction density [42]. An elevated concentration of BDM residues enriches dominant microorganisms, which respond to environmental fluctuations by enhancing their interplay. Zhao et al. demonstrated that BDM significantly increased rhizosphere microbial diversity, enriched beneficial taxa, and enhanced microbial network complexity and stability, ultimately leading to improved peanut yield [43]. Composting may modify the network’s topological structure and enhance the complexity and density of interactions among microbial communities, which is reflected in the reduced nodes and expanded edges in the P 0.1% and P 1% treatments. The enhanced proportion of positive correlations in the P 1% treatment signifies a heightened symbiotic pattern among bacterial populations, reflecting both the centralization of microbial consortium composition and the intensification of interspecies interactions [44]. These results indicate that the richer nutrient supply might promote sophistication in symbiotic networks.

#### 2.3.3. Sensitive Bacterial Populations

In order to identify the key species influencing environmental changes, bacterial populations sensitive to different treatments were identified using LEfSe (Figure S1). A total of 77 biomarkers were monitored across the 10 groups, based on the principle of marked differentiation (LDA ≥ 2) at various taxonomic levels. Across all R treatments, the number of biomarkers remained below five. Specifically, 16, 3, and 6 bacterial populations were found to be sensitive to the introduction of residues within the M 0.01%, M 0.1%, and M 1% treatments, respectively. In addition, the *Ramlibacter* and *Bradyrhizobium* genera detected in the M 1% treatment have been reported to exhibit BDM degradation potential [45]. In further studies, these potential functional microorganisms could be isolated and validated through pure culture experiments to elucidate their roles in PBST-BDM degradation process. The number of biomarkers in the P 1% treatment reached 40, indicating a high variability in the bacterial community. *Sporocytophaga,* detected in the P treatment, demonstrates cellulose-degrading capabilities that are intrinsically linked to composting processes [46]. The distinct biomarkers identified through LEfSe analysis among treatment groups suggest that specific microbial taxa may play pivotal roles in composting and PBST-BDM biodegradation processes.

### 2.4. Responses of Soil Metabolism to the Treatments of PBST Raw Material, PBST Residues, and PBST Composting Product

#### 2.4.1. Advanced Multivariate Evaluation of Metabolites

**Figure 5:**
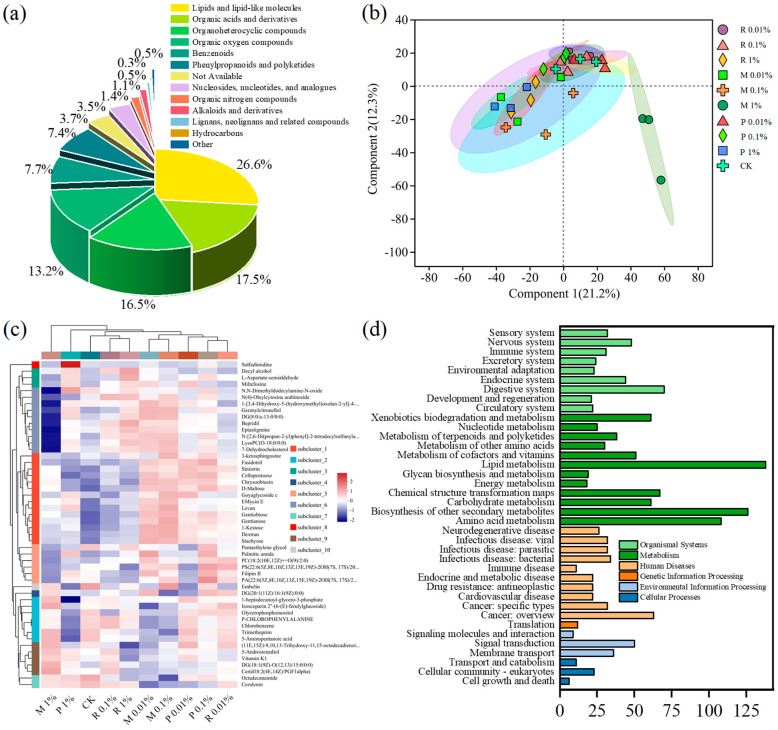
Metabolite classification and proportion (**a**), PLS-DA scores plot of metabolites (**b**), cluster of metabolites (**c**), and metabolic pathway involved in different treatments (**d**).

Metabolomic investigation was performed to ascertain the impact of non-indigenous organisms on soil metabolic activity (Figure 5a). Soil metabolites are primarily categorized into 13 groups based on the HMDB database. Lipids and lipid-like molecules comprised 26% of the primary types of metabolites. PLS-DA was applied to scrutinize the disparities between CK and treatments intra- and inter-group, evaluating the impacts of various treatments on soil metabolites (Figure 5b). Evidently, all treatments exhibit similar metabolites, except for the notable difference between the M 1% treatment and CK group, indicating significant dissimilarity in soil metabolites between the M 1% treatment and CK. Furthermore, a significant contrast was observed between the M 1% treatment and other treatments, implying that the alteration in soil metabolites is influenced by PBST-BDM residues. The model’s explanatory degree is 21.2% and 12.3%. By clustering the differentially expressed metabolites, the changing patterns of various metabolite groups can be visually observed (Figure 5c). The metabolite cluster analysis yielded results consistent with PLS-DA (*p* < 0.05). Exogenous substances have the potential to perturb soil metabolites, and this response may manifest in the composition and quantity of metabolites [47]. Previous study has reported that organic fertilizer usage can modify metabolic processes in microbial communities, increasing the bioavailability of soil amino acids for microbial and plant absorption [48]. The governance of amino acid metabolism serves as an adaptive strategic response of organisms to environmental stress, according to Zhang’s study [49]. After exposure to oxytetracycline and MPs, various metabolites, such as organic acids and sugars, underwent significant changes [50]. Sugars and organic acids were identified as crucial factors influencing rhizosphere microbial communities [51]. One study noted profound variations in soil metabolites resulting from BDM residues, with a higher quantity of differential metabolites compared to LDPE film residues [7].

Pathway enrichment analysis using KEGG indicated that the implicated pathways predominantly pertained to metabolism (1.18–9.07%), organismal systems (1.38–4.60%), human diseases (0.72–4.14%), and environmental information processing (0.59–3.29%) (Figure 5d). The key pathways at Level 2 within metabolism included lipid metabolism, the biosynthesis of other secondary metabolisms, amino acid metabolism, and so on. The digestive system, nervous system, and endocrine system emerged as the primary pathways within the organismal systems cluster. Cancer overview and signal transduction were the main pathways within human diseases and environmental information processing, respectively. The various pathways may represent the responses of different microorganisms to environmental changes. For instance, the high prevalence of amino acid metabolism indicates that it could function as an energy and carbon source for microorganisms, facilitating their proliferation and metabolic activities [52].

#### 2.4.2. Soil Differential Metabolites Analysis

The volcano plot alongside the classification of significant differential metabolites is presented in Figure S3. The critical differential metabolites were structured into six distinct groups. The R 0.01%, R 0.1%, and R 1% treatments significantly influenced 59, 42, and 187 metabolites, respectively. Noticeable changes were observed in the first five soil metabolite classifications. Substantial differences in the differential metabolites were monitored across the three M treatments that involved adding PBST fragments, indicating a strong influence of PBST residues on soil metabolites. In comparison to CK, 281, 162, and 214 metabolites were identified in M 0.01%, M 0.1%, and M 1% treatments, respectively. The metabolites showing significant changes were distributed across six classifications under C treatments. In P 0.01%, P 0.1%, and P 1% treatments, a total of 100, 151, and 114 differential metabolites were detected, respectively. Among them, the first four metabolite classifications showed more significant changes.

Heightened presence of differential metabolites in the M Group may be associated with the composition of BDM. The aromatic ring in terephthalic acid, an essential component of BDM, leads to an increase in benzenoids and organoheterocyclic compounds [53]. Lipids and lipid-like molecules are generated through the stimulation of butylene adipate, a lipid-related substance [54]. Terephthalic acid in BDM is likely to elevate the levels of organic acids. Further investigation is warranted to comprehend the prospective consequences of BDM residues on soil metabolite structures.

#### 2.4.3. Differential Pathway Enrichment

Exploration of differential pathway enrichment was conducted (Figure S4). While organismal systems and human diseases emerged as the primary pathways, their prevalence varied across treatments. Analysis revealed distinct enrichment pathways for three, nine, and eight organismal systems and two, five, and five human diseases at R 0.01%, R 0.1%, and R 1%, respectively. Both in M 0.01%, M 0.1%, and M 1% treatments, eight organismal systems were observed, with slight differences in the abundance of human diseases. P 0.01% treatment showed 6 metabolisms and 4 human diseases, while both P 0.1% and P 1% treatments exhibited 10 organismal systems and 5 human diseases. Variations in pathways suggest that microorganisms respond differently to treatment conditions. For instance, PBST-BDM residues may serve as energy sources for soil microorganisms, leading metabolites and metabolic pathway changes. Compost products, rich in nutrients, impact soil reflected in differences in metabolites and metabolic pathways.

#### 2.4.4. Comprehensive Effects of Soil Physicochemical Properties, Microbial Communities, Enzyme Activities, and Microbial Metabolism on Chinese Cabbage Growth

**Figure 6:**
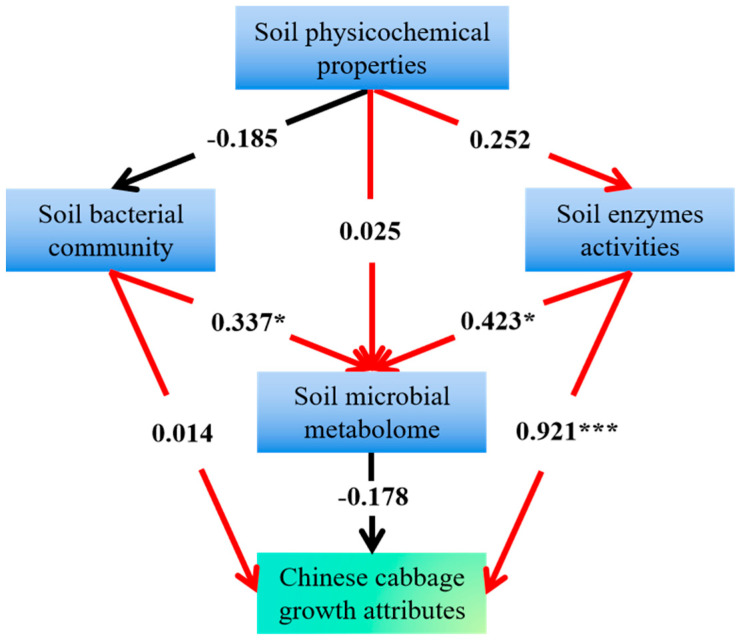
The structural equation among soil physicochemical properties, soil microbial community, enzyme activities, and soil microbial metabolome on growth attributes of Chinese cabbage. Note: ‘*’ represents *p* < 0.05, and ‘***’ represents *p* < 0.001.

Structural equation modeling (SEM) was conducted in this study to investigate the comprehensive effects of soil physicochemical properties, microbial communities, enzyme activities, and microbial metabolism on Chinese cabbage growth (Figure 6). The results showed that soil physicochemical properties (path coefficient 0.025), soil enzyme activities (0.423), and the microbial community (0.337) primarily indirectly affect Chinese cabbage growth by regulating the soil microbial metabolism, consistent with previous studies [55,56]. Soil enzyme activity is a core factor driving soil microbial metabolism, indirectly promoting crop growth by facilitating organic matter decomposition, nutrient cycling, and improving soil physicochemical properties [57]. Notably, soil enzyme activities exerted a highly significant direct positive effect on Chinese cabbage growth (path coefficient 0.921). However, the direct effect of the microbial community on Chinese cabbage growth was not significant (path coefficient 0.014), contrasting with previous studies emphasizing its crucial role in crop growth. For instance, Gao et al. (2023) reported a positive correlation between the increased abundance of specific taxa (e.g., *Bacillus*) and enhanced crop growth [58]. The non-significant direct effects observed in this study are likely attributable to the absence or non-dominance of key functional groups (e.g., growth-promoting bacteria) in the soil and/or environmental constraints on microbial functionality. These results imply that the direct contribution of microbial communities to crop growth is likely limited, highlighting the critical role of environmental conditions [59]. Future studies should integrate metagenomics with functional validation experiments to elucidate the specific mechanisms through which microbial communities influence Chinese cabbage growth.

## 3. Materials and Methods

### 3.1. Soil Preparation and Experiment Materials

Soil was sourced from a farm located in Beijing, China, air-dried and sieved through a 2 mm mesh. The type of soil texture is loam. The basic initial properties of the soil included a pH value of 7.20, an electrical conductivity (EC) of 85 μS/cm, a bulk density of 1.42 g/cm^3^, and an organic matter content of 9.18 g/kg. This site was free from any past use of PE or BDM. The materials were provided by Sinopec Beijing Research Institute of Chemical Industry, including PBST-BDM raw material (PBST resin granules), BDM with the main ingredient of PBST (produced via blow molding), and the composting product of PBST (bio-composted at 58 °C for 180 days). The carbon content of the mulch film was 61.0%, which decreased to 42.9% after composting. The density of PBST-BDM is 1.35 g/cm^3^, and the material was cut into fragments of 1 mm × 1 mm, 5 mm × 5 mm, and 10 mm × 10 mm to match BDM residues.

### 3.2. Experiment Procedure

Pot trials were performed in the glasshouse. Chinese cabbage (*Brassica rapa* subsp. *pekinensis*) was selected as the model plant due to its relatively short growth period (60 days to maturity), facilitating pot-based experimentation, and comparative sensitivity to soil environmental stressors, such as microplastics [60]. Ceramic vessels with dimensions of 45 cm × 23 cm × 17 cm (length × width × height), each filled with 15 kg of soil, were employed to plant Chinese cabbage. In addition to the control group (soil only), we established nine treatments with three replicates each (Table S1), using three concentration gradients (0.01%, 0.1%, and 1%). These treatments represent PBST-BDM in its three typical agricultural states-raw material input, residue accumulation, and composting product-to simulate different exposure scenarios. The study aims to clarify how PBST-BDM at different stages affects Chinese cabbage growth and soil properties while exploring practical application methods to promote sustainable agricultural production. The ceramic vessels were arranged randomly, and their positions were rotated every two weeks to minimize potential micro-environmental biases. The temperature and humidity data were recorded at 10 min intervals throughout the experiment, which are shown in Figure S5. Although a total of 100 premium Chinese cabbage seeds were planted, the number of plants was reduced to 10 on the eighth day after sowing to guarantee a suitable density. No fertilizers were applied to maintain a controlled soil micro-environment. After sowing, daily irrigation was applied uniformly across all treatments to ensure consistent management.

### 3.3. Analytical Methods

#### 3.3.1. Growth Parameters of Chinese Cabbage

The emergence rate was quantified by the number of surviving seeds/sowing amount after sowing for 3 days. Plant specimens were photographed before the first true leaf emergence for subsequent ImageJ-based (Version v1.53t) cotyledon area measurement. Plant height, leaf area, and fresh biomass production were also examined (7 days/time).

#### 3.3.2. Soil Physicochemical Properties

Five-point sampling methodology was conducted to acquire soil samples with three duplicates for all treatments when the Chinese cabbages were harvested. EC and pH were obtained by a multiparameter water quality meter (SevenExcellenceS400, Mettler-Toledo, Zurich, Switzerland). The organic carbon (SOC) content was determined using the potassium dichromate method, while available phosphorus (AP) was extracted with 0.5 M sodium bicarbonate (NaHCO_3_, pH 8.5) and quantified via spectrophotometry [7]. The elemental analyzer was utilized to assess total carbon (TC) and total nitrogen (TN) (Flash Smart NC SOIL, Beijing, China). The activities of urease (UE), peroxidase (POD), and alkaline phosphatase (AKP) were ascertained based on the manufacturer’s protocols of kits [61]. Concisely, 0.1 g of soil was combined with different enzyme extractants in a test tube. The mixture was centrifuged after thorough extraction, followed by the introduction of the reaction solution. The enzyme concentration was determined by measuring the absorbance of the supernatant at a specified wavelength using spectrophotometry.

### 3.4. Microbiological Analysis

Soil samples of CK, R 0.01%, R 0.1%, R 1%, M 0.01%, M 0.1%, M 1%, P 0.01%, P 0.1%, and P 1% treatments were gathered with three duplicates after Chinese cabbage was harvested (stored at −80 °C before analysis). All samples designated for microbiological analysis were dispatched to Shanghai Majorbio Technology Co., Ltd. (Shanghai, China) for the high-throughput sequencing of the 16S rRNA gene using the Illumina MiSeq platform (Illumina, USA). Prior to sequencing, DNA was extracted using the E.Z.N.A.^®^ Soil DNA Kit (Omega Bio-tek, Norcross, GA, USA). The V3-V4 hypervariable regions were amplified via PCR with primers 338F (5′-ACTCCTACGGGAGGCAGCAG-3′) and 806R (5′-GACTACHVGGGTWTCTAAT-3′), followed by the purification of the amplified products using the PCR Clean-Up Kit (YuHua, Shanghai, China). The paired-end raw sequencing reads were subjected to quality control using fastp (version 0.19.6), followed by assembly with FLASH (version 1.2.11). During processing, reads were truncated at positions where the average quality score fell below 20 within a 50 bp sliding window. Reads shorter than 50 bp after truncation were discarded. Overlapping sequences longer than 10 bp were merged based on their overlapping regions, with a maximum mismatch ratio of 0.2 allowed in the overlap. Operational taxonomic units (OTUs) were clustered from sequences at a 0.03 distance threshold (corresponding to 97% similarity) using UPARSE 7.1. The number of 16S rRNA gene sequences from each sample was rarefied to 20,000 after the quality filtering of raw sequences, which still yielded an average Good’s coverage index of 99.09%.

### 3.5. Non-Specific Soil Metabolic Profiling

Soil samples of CK, R 0.01%, R 0.1%, R 1%, M 0.01%, M 0.1%, M 1%, P 0.01%, P 0.1%, and P 1% treatments were taken and stored at −80 °C until analysis. Before being analyzed through LC-MS/MS, soil samples were extracted after frozen tissue grinding (−10 °C, 50 Hz, 6 min) and low-temperature ultrasonic extraction (30 min). All samples were mixed in equal volumes to create pooled quality control samples (QC) to monitor the stability of the analysis. Both QC and analytical samples were processed under identical conditions. The LC-MS/MS analysis of the samples was conducted on a Thermo UHPLC-Q Exactive HF-X system equipped with an ACQUITY HSS T3 column (100 mm × 2.1 mm i.d., 1.8 μm; Waters, Milford, MA, USA). The mobile phases consisted of 0.1% formic acid in water-acetonitrile (95 : 5, *v*/*v*) (solvent A) and 0.1% formic acid in acetonitrile-isopropanol-water (47.5 : 47.5 : 5, *v*/*v*) (solvent B). The flow rate was 0.40 mL/min and the column temperature was 40 °C. The injection volume was 3 μL. The mass spectrometric data were collected using a Thermo UHPLC-Q Exactive HF-X Mass Spectrometer (Thermo Fisher Scientific, Waltham, MA, USA) equipped with an electrospray ionization (ESI) source operating in positive mode and negative mode. The detection was carried out over a mass range of 70–1050 *m*/*z*. Progenesis QI (Waters Corporation, Milford, CT, USA) software (Version v4.1) was employed to perform the primary data and exported in the CSV format. The identification of metabolites involved querying a database to acquire the data matrix, which was then analyzed utilizing Majorbio cloud platform. Differential metabolites between two groups were mapped into their biochemical pathways through metabolic enrichment and pathway analysis based on KEGG database. Metabolite classification, clustering, and KEGG metabolic pathway analysis were conducted by metabolic enrichment methods, with visualization performed utilizing Origin software (Version 9.8.0.200).

### 3.6. Statistical Assessment

The variations in soil physicochemical properties across treatments were assessed using one-way ANOVA with Tukey’s Honestly Significant Difference (HSD) test (*p* < 0.05). The MajorBio platform (http://www.majorbio.com/) was utilized for analyzing the bacterial community characteristics. The assessment of bacterial diversity at the OTU level was conducted via the Shannon index. Bray-Curtis dissimilarity was utilized in principal coordinate analysis (PCoA) to ascertain the similarity between bacterial populations across multiple samples. Histograms and heat maps were utilized to illustrate the variability of bacterial composition. The biomarkers were identified using the LEfSe method, which includes detecting significantly differentially abundant species across groups via the Kruskal-Wallis test, validating their subgroup consistency through the Wilcoxon rank-sum test, and quantifying their discriminative impact on group separation with linear discriminant analysis (LDA). Metabolite clustering was demonstrated using a cluster heat map. Metabolites exhibiting significant differences were identified by VIP > 1 and *p* < 0.05 using Variable Importance in Projection (VIP) obtained from the PLS-DA model and *p*-values from Student’s *t*-test. The volcano plot displayed differential metabolites across all groups. Mantel test analysis was conducted using the R (Version 4.3.1) package “linkET” and “ggplot2”, and correlation calculations used Spearman correlation coefficients. The visualization of the co-occurrence network of microbial species was exhibited using software, including R language (Version 4.3.1) and Gephi (Version 0.10.1). Python (Version 1.20) package “scipy.stats” was used to perform enrichment analysis for experimental treatments.

## 4. Conclusions

This study elucidates the differential effects of PBST-BDM raw material (R), PBST-BDM residues (M), and PBST-BDM composting product (P) on both crop growth and soil quality. The findings suggest that M treatments had a notably detrimental influence on Chinese cabbage growth, with the emergence rate, plant height, leaf area, and biomass accumulation of Chinese cabbage being decreased by 30.4% (*p* < 0.05), 2.71 cm (*p* < 0.05), 39.0% (*p* < 0.05), and 1.86 g (*p* < 0.05), respectively, in the M 1% treatment (compared to the CK). In general, PBST-BDM residues affect the activities of microorganisms directly or by altering the soil properties. A positive effect on both Chinese cabbage and soil was observed after PBST-BDM composting, and, compared to the CK, increases of 8.89% (*p* < 0.05), 4.96 cm (*p* < 0.05), 36.3% (*p* < 0.05), and 2.31 g (*p* < 0.05) were detected, respectively, in the P 1% treatment. The topological structure of the microbial network indicates a stronger symbiotic pattern between species after composting. Specific composting conditions accelerate the early breakdown of PBST-BDM residues, alleviating the adverse ecological impacts. While composting PBST-BDM shows promise in mitigating its negative impacts on crop growth and microbial ecology, further studies across diverse soil types and climatic conditions are needed to evaluate decomposition rates and residual effects for broader applicability. This study was conducted under controlled conditions and has limited applicability to real-world agricultural systems. Future research should focus on long-term, systematic field investigations to generate more practical foundational data. In addition, the future quantification of organic carbon release from plastic degradation and its relationship with plant-derived carbon would provide critical insights into soil-microbe-plant carbon cycling.

PBST-BDM involves ensuring food security production and long-term soil safety in farmland. In-depth research should investigate the tolerance of diverse plant species to PBST-BDM residues and address long-term soil health risks from residual PBST-BDM accumulation. It is also critical to clarify the spatiotemporal variability in PBST-BDM degradation efficiency across climates and soil types, as inconsistent degradation rates may exacerbate microplastic pollution. In the case of PBST-BDM residues, composting serves as an eco-friendly and sustainable treatment approach. Through this process, PBST-BDM residue is decomposed by microorganisms that utilize it as their energy source. Additional research is warranted to comprehend the influence of composting on elemental cycling throughout the entire ecosystem. At present, the use of BDM indeed poses challenges related to environmental/ecological issues and imperfect regulatory systems. To ensure ecological safety, it is imperative to establish standardized certification systems for BDM degradation performance to regulate market quality, along with policy support to dismantle technological monopolies.

## Data Availability

Data are contained within the article.

## Supplementary Materials

The following supporting information can be downloaded at: https://www.mdpi.com/article/10.3390/plants14131902/s1, Figure S1: LEfSe cladogram of soil bacterial community; Figure S2: Co-occurrence network of soil bacterial community based on correlation analysis; Figure S3: Volcano plot of differential metabolites for R 0.01% (a), R 0.1% (b), R 1% (c), M 0.01% (d), M 0.1% (e), M 1% (f), P 0.01% (g), P 0.1% (h), and P 1% (i) treatments; Figure S4: KEGG pathway enrichment analysis; Figure S5: The temperature (a) and air humidity (b) during the experiment; Table S1: Experimental design; Table S2: Significant differences in plant growth parameters among treatments (*p* < 0.05).; Table S3: Co-occurrence network properties of various treatments in soil microbial communities.
