# Supplementary material for: Composted PBST Biodegradable Mulch Film Residues Enhance Crop Development: Insights into Microbial Community Assembly, Network Interactions, and Soil Metabolism
Source: Plants (Basel). 2025 Jun 20;14(13):1902. doi: 10.3390/plants14131902 (PMC12252030; doi:10.3390/plants14131902)
Supplement: Supplementary file 1 [file plants-14-01902-s001.zip › plants-3623483-supplementary.pdf]

Supporting Information

Composted PBST Biodegradable Mulch Film Residues Enhance Crop Development: Insights into Microbial Community Assembly, Network Interactions, and Soil Metabolism

Liuliu Li [1,†], Liyuan Liu [2,†], Guoyuan Zou [1,*], Xuexia Wang [1], Li Xu [3], Yong Yang [4], Jinfeng Liu [4], Huabo Liu [5], Dongsheng Liu [1,*]

[1] Institute of Plant Nutrition, Resources and Environment, Beijing Academy of Agriculture and Forestry Sciences, Beijing 100097, China; liliuhb@126.com (L.L.); wxx0427@163.com (X.W.)
[2] Institute of Environment and Sustainable Development in Agriculture, Chinese Academy of Agricultural Sciences, Beijing 100081, China; liuliyuan1001@163.com (L.L.)
[3] Institute of Quality Standard and Testing Technology, Beijing Academy of Agriculture and Forestry Sci-ences, Beijing, 100097, China; xuliforever@163.com (L.X.)
[4] SINOPEC (Beijing) Research Institute of Chemical Industry Co., Ltd, Beijing 101111, China; yangy.bjhy@sinopec.com (Y.Y.); liujinf.bjhy@sinopec.com (J.L.)
[5] Miyun District Agricultural Technology Extension Station in Beijing City, Beijing 101599, China; liuhuabo@163.com (H.L.)
[*] Correspondence: gyzou@163.com; LLSLDS@163.com; Tel.: +86-010-5150-7539 (D.L.)
[†] Both authors contributed equally to this work.

Table S1. Experiment design.

Treatments		Addition type	Soil weight (kg)	Weight ratios between additions and soil (%)
Control	CK	No addition	7.5	0.0
Raw materials (R)	R 0.01%	PBST	7.5	0.01
	R 0.1%	PBST	7.5	0.1
	R 1%	PBST	7.5	1
Mulch film (M)	M 0.01%	PBST–BDM fragments	7.5	0.01
	M 0.1%	PBST–BDM fragments	7.5	0.1
	M 1%	PBST-BDM fragments	7.5	1
Compost product (P)	P 0.01%	Compost products of PBST	7.5	0.01
	P 0.1%	Compost products of PBST	7.5	0.1
	P 1%	Compost products of PBST	7.5	1

Table S2. Significant differences in plant growth parameters among treatments ($p < 0.05$).

Treatments	Emergence rate	Plant height	Leaf area	Biomass accumulation
CK	d	c	c	e
R 0.01%	e	b	c	d
R 0.1%	f	b	d	d
R 1%	g	b	d	c
M 0.01%	c	b	b	c
M 0.1%	b	b	b	b
M 1%	a	a	a	a
P 0.01%	g	c	d	f
P 0.1%	h	d	d	f
P 1%	h	d	e	f

Table S3. Co-occurrence network properties of various treatments in soil microbial communities.

	CK	R 0.01%	R 0.1%	R 1%	M 0.01%	M 0.1%	M 1%	P 0.01%	P 0.1%	P 1%
Number of nodes	25	18	19	24	24	24	40	30	23	22
Number of links	117	66	85	147	112	145	303	193	121	133
Positive edges (%)	61.5	69.7	62.4	59.2	69.6	64.1	56.8	61.1	55.4	72.9
Average degree	9.36	7.33	8.95	12.3	9.33	12.1	15.2	12.9	10.5	12.1
Average clustering coefficient	0.93	0.88	0.93	0.96	0.94	0.96	0.97	0.96	0.95	0.95

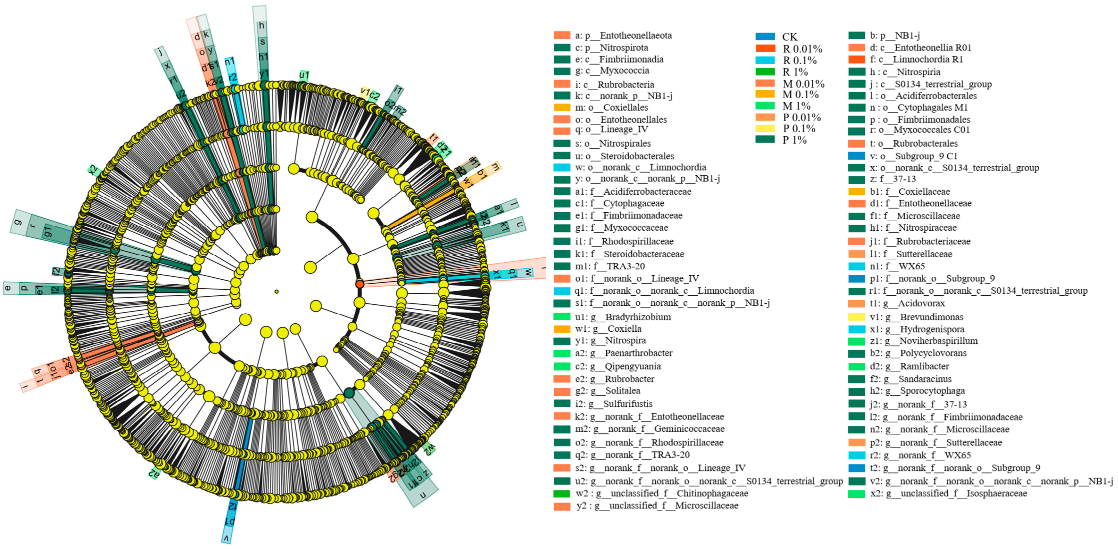

Figure S1. LEfSe cladogram of soil bacterial community. R 0.01%, R 0.1% and R 1% represent the weight ratios between PBST raw material and soil; M 0.01%, M 0.1% and M 1% represent the weight ratios between PBST-BDM fragments and soil; P 0.01%, P 0.1% and P 1% represent the weight ratios between PBST-BDM composting and soil.

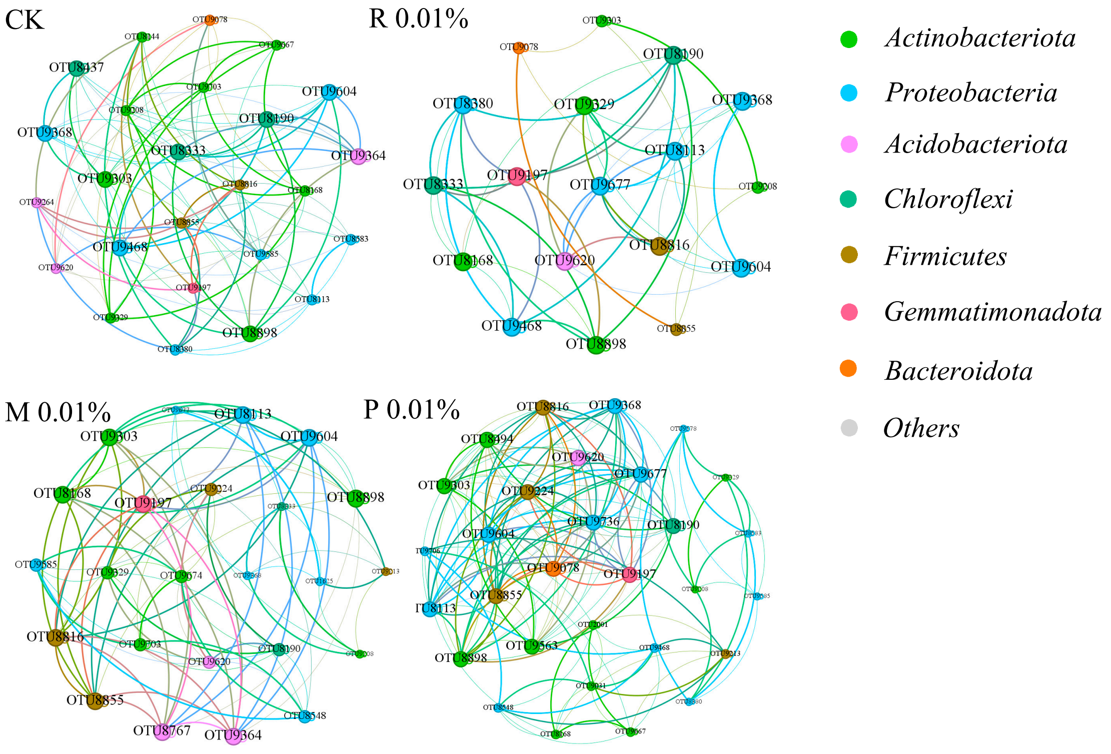

Figure S2. Co-occurrence network of soil bacterial community based on correlation analysis. Networks were constructed at operational taxonomic unit (OTU) level. The sizes of nodes (OTU) were scaled to the degree of nodes and the colour of the node were colored on phylum level. The edges colored by red and green represent positive and negative correlations, respectively. The significant difference between groups based on network Topological indices (*: $p < 0.05$).

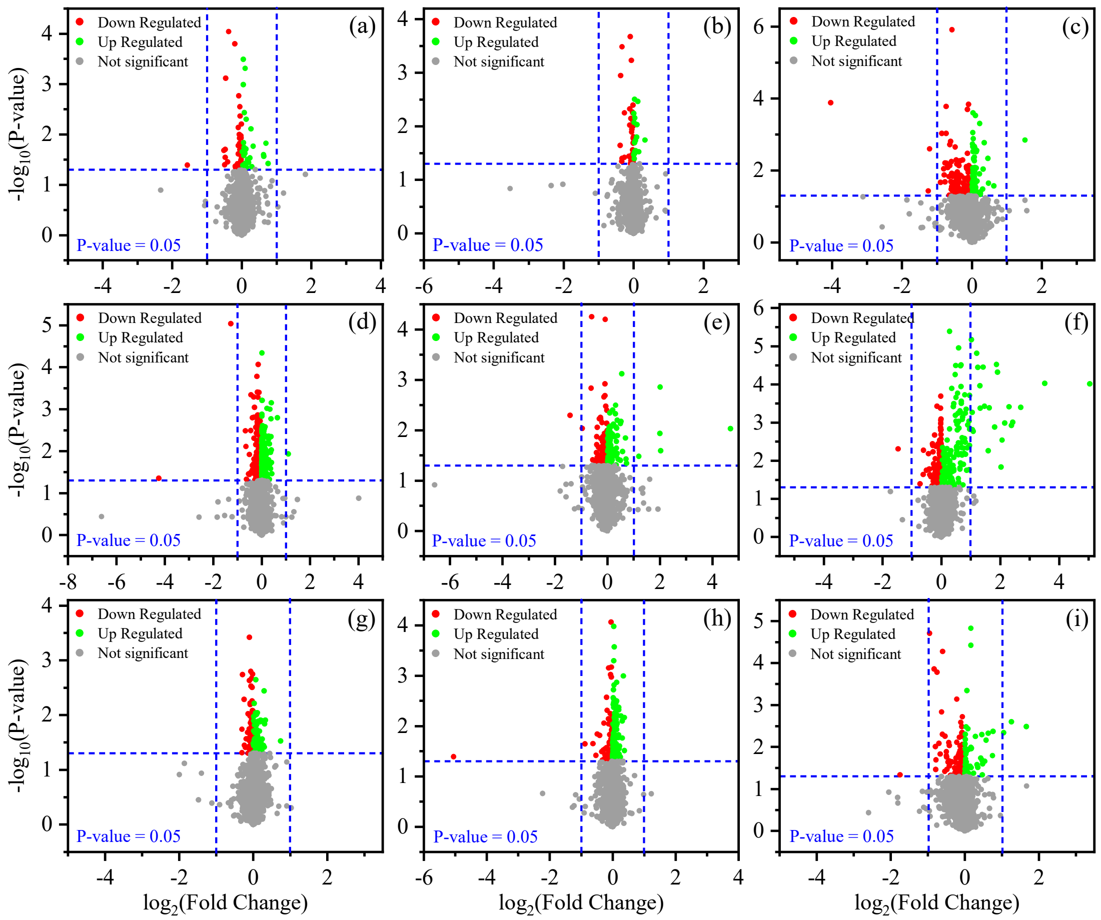

Figure S3. Volcano plot of differential metabolites for R 0.01% (a), R 0.1% (b), R 1% (c), M 0.01% (d), M 0.1% (e), M 1% (f), P 0.01% (g), P 0.1% (h) and P 1% (i) treatments.

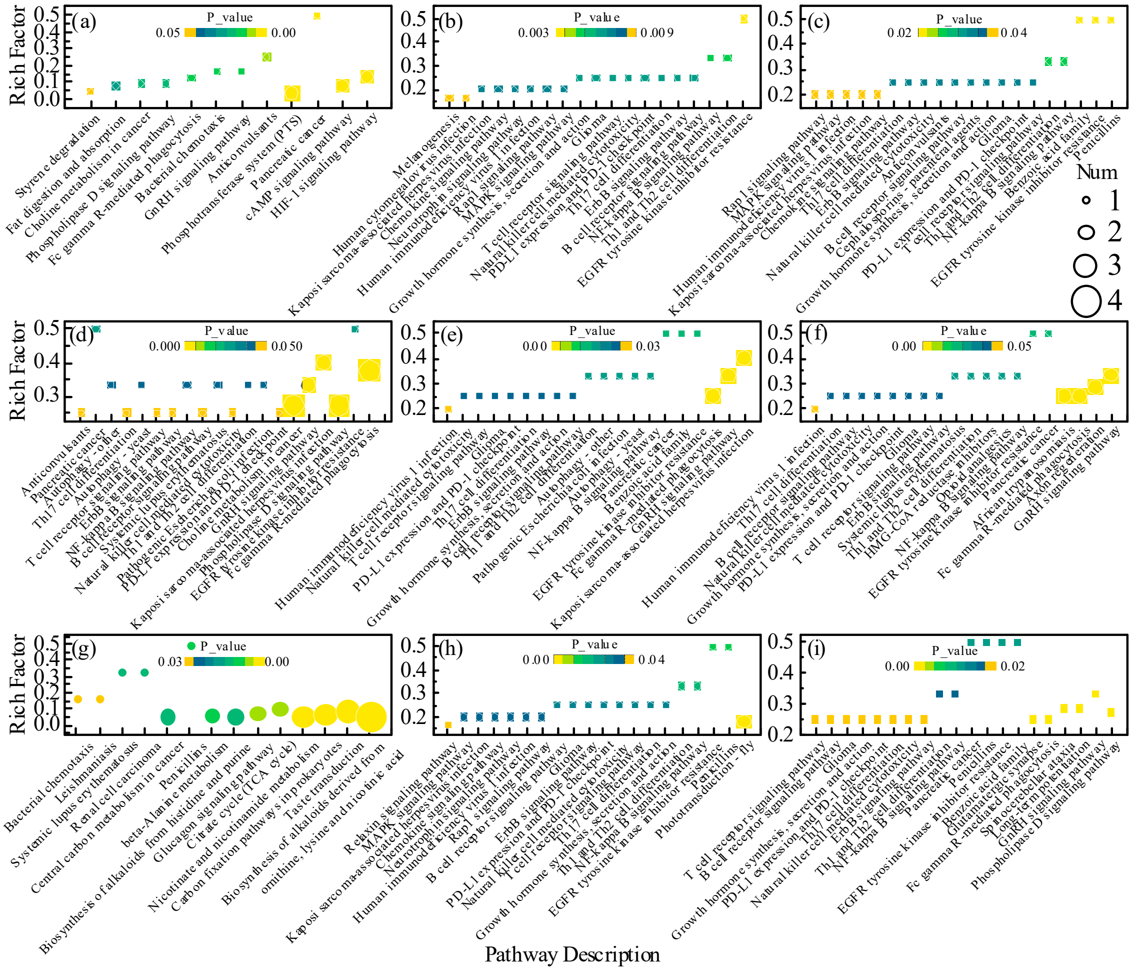

Figure S4. KEGG pathway enrichment analysis. (a) CK vs R 0.01%, (b) CK vs R 0.1%; (c) CK vs R 1%; (d) CK vs M 0.01%; (e) CK vs M 0.1%; (f) CK vs M 1%; (g) CK vs P 0.01%; (h) CK vs P 0.1%; (i) CK vs P 1%.

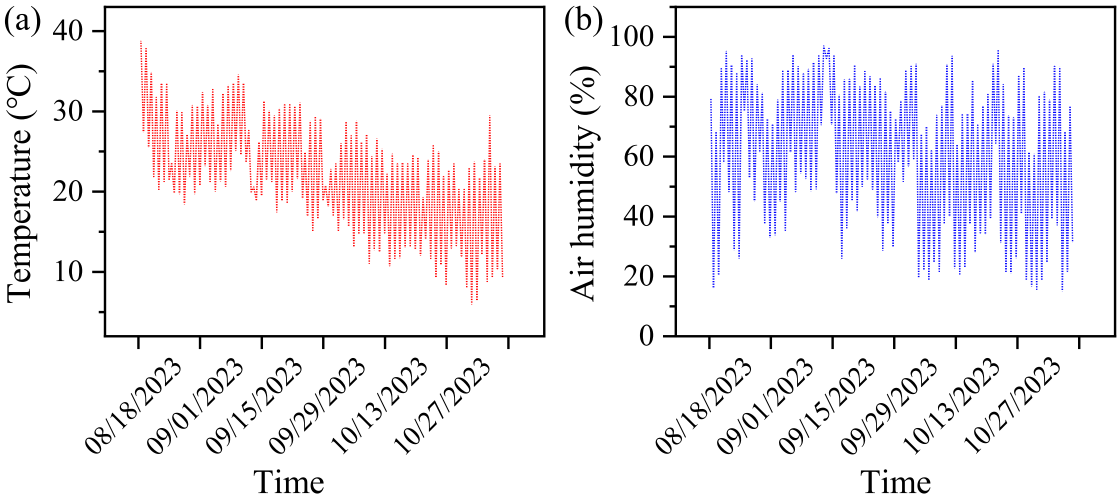

Figure S5. The temperature (a) and air humidity (b) during the experiment
